# Network dynamics in the healthy and epileptic developing brain

**DOI:** 10.1162/NETN_a_00026

**Published:** 2018-03-01

**Authors:** Richard Rosch, Torsten Baldeweg, Friederike Moeller, Gerold Baier

**Affiliations:** Wellcome Trust Centre for Neuroimaging, University College London, United Kingdom; Developmental Neurosciences Programme, UCL Great Ormond Street Institute of Child Health, University College London, United Kingdom; Department of Clinical Neurophysiology, Great Ormond Street Hospital, London, United Kingdom; Cell and Developmental Biology, University College London, United Kingdom

**Keywords:** EEG, Dynamic network, Epilepsy, State transitions, Computational analysis

## Abstract

Electroencephalography (EEG) allows recording of cortical activity at high temporal resolution. EEG recordings can be summarized along different dimensions using network-level quantitative measures, such as channel-to-channel correlation, or band power distributions across channels. These reveal network patterns that unfold over a range of different timescales and can be tracked dynamically. Here we describe the dynamics of network state transitions in EEG recordings of spontaneous brain activity in normally developing infants and infants with severe early infantile epileptic encephalopathies (*n* = 8, age: 1–8 months). We describe differences in measures of EEG dynamics derived from band power, and correlation-based summaries of network-wide brain activity. We further show that EEGs from different patient groups and controls may be distinguishable on a small set of the novel quantitative measures introduced here, which describe dynamic network state switching. Quantitative measures related to the sharpness of switching from one correlation pattern to another show the largest differences between groups. These findings reveal that the early epileptic encephalopathies are associated with characteristic dynamic features at the network level. Quantitative network-based analyses like the one presented here may in the future inform the clinical use of quantitative EEG for diagnosis.

## INTRODUCTION

Epilepsy is the most common primary neurological disorder globally, with a particularly high incidence in infancy and childhood (Olafsson et al., 2005). In a group of epilepsy syndromes, the burden of epileptic discharges can cause severe, persistent brain dysfunction, that is, a recognizable encephalopathy. When these occur in early infancy, they are known as early infantile epileptic encephalopathies (EIEE) (Ben-Ari & Holmes, 2006; Jette et al., 2015). Within the category of severe epilepsies, there are several discrete electroclinical syndromes that follow specific developmental timelines, occurring mainly in the neonatal period or very early infancy (e.g., Ohtahara syndrome), later during infancy (e.g., infantile spasms / West syndrome), or in early childhood (e.g., Lennox-Gastaut syndrome). This developmental pattern can also be observed in individual patients, such that a syndromic pattern may evolve, such as from Ohtahara to West syndrome during development. This suggests that despite an individually persistent cause for the epilepsy (such as a genetic mutation or structural lesion), it is specific stages of brain development that translate the abnormality into age-specific, recognizable electroclinical phenotypes (Kodera et al., 2016; Ohtahara & Yamatogi, 2006).

Electroencephalography (EEG) gives a rich picture of dynamic neuronal function and regionally distinct oscillatory brain behavior in frequency ranges that span several orders of magnitude (Lopes da Silva, 1991). In clinical practice, EEG analysis is focused on visual pattern recognition of specific waveform abnormalities (e.g., epileptiform discharges) associated with specific clinical correlates (e.g., increased risk of epileptic seizures). Visual analysis—while essential—is biased towards certain observable features: For example, between-channel correlation of low-frequency, high-amplitude discharges is much more readily apparent than that of high-frequency, low-amplitude discharges. Quantitative, automatic analysis may reveal some of these EEG features usually overlooked by visual analysis alone (Tong & Thakor, 2009).

Graph theory, or network-based approaches to understanding neuronal function in terms of functional networks, have recently emerged in imaging neuroscience. Particularly in functional magnetic resonance imaging (fMRI) of the resting state, this has led to the discovery that neuronal networks show functionally relevant and quantifiable fluctuation between different constellations, or states over time (Allen et al., 2014; Krienen, Yeo, & Buckner, 2014). Similar methods based on graph theory have now been applied to electrophysiological signals from EEG and MEG (magnetoencephalography) recordings in humans (Boersma et al., 2011; Brookes et al., 2011; Maldjian, Davenport, & Whitlow, 2014), and suggest that the high temporal resolution in these signals can be harnessed to identify recognizable microstates at millisecond-to-second timescales and characterize the switching between them (Baker et al., 2014; Khanna, Pascual-Leone, Michel, & Farzan, 2015; Koenig et al., 2002; Van De Ville, Britz, & Michel, 2010; Vidaurre et al., 2016).

Dynamic features not directly visible in EEG analysis—such as the microstate dynamics described above—are not commonly considered in the analysis of clinical EEG recordings. There is an emerging literature on the computational analysis of EIEE phenotypes (Japaridze et al., 2013; Japaridze et al., 2016) and related abnormal EEG patterns (Ching, Purdon, Vijayan, Kopell, & Brown, 2012; Liu & Ching, 2017; Zubler et al., 2014). But our understanding of intrinsic network dynamics in these phenotypes is still limited. Yet, these network dynamics are potentially important to understand whole-brain dysfunction as seen in the EIEEs, where there is often not a sharp distinction between seizure patterns and interictal abnormalities.

We build on the existing literature by evaluating simple quantitative summary measures of the network dynamics in spontaneous brain activity in the healthy and epileptic developing brain. A formally related approach has previously been introduced by Betzel et al. (2012), where temporal similarity/dynamics matrices were calculated for specific frequency bands for short EEG segments. We further evaluate the relationship between these correlation-based network dynamics and power distribution in early infantile EEG.

The work presented in this paper thus has two main goals: (a) to describe a method to quantify network dynamics in terms of dynamical switches between EEG states based on correlation patterns and power distributions, thus deriving a multivariate feature space capturing network-level brain dynamics; and (b) to evaluate whether this approach captures pathological brain dynamics by mapping two distinct EIEE electroclinical syndromes (Ohtahara syndrome, West syndrome) onto this brain dynamics feature space. In the future, such an approach may prove valuable for resolving diagnostic uncertainties (e.g., in neonatal epilepsy), but it may also inform computational models of neuronal populations, and thus help identify the neurobiological mechanisms underlying the phenotypes seen in this group of severe epilepsies.

## METHODS

### Subjects

This study is focused on establishing estimates that describe EEG microstate dynamics using different network measures, and thus illustrates the methodology on a small number of participants with profound EEG abnormalities. Both patients and control EEGs were selected from previously recorded standard pediatric clinical EEGs. The selection was based on classification by a clinical neurophysiologist with expertise in pediatric EEG (FM).

**Figure 1.**
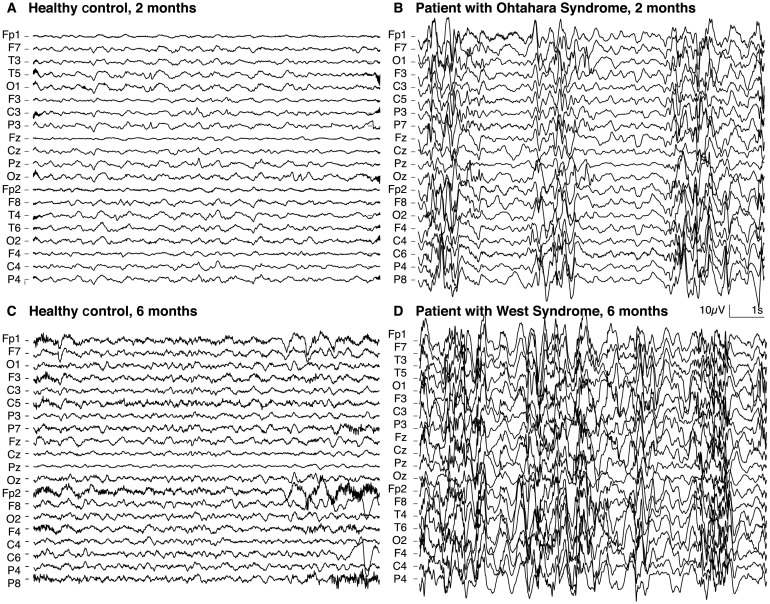
EEG recordings of healthy controls (A, C) and patients with EIEEs (B, D). Compared with healthy controls at 2 months (A), Ohtahara syndrome is associated with abnormal burst-suppression patterns disrupting the ongoing background, characterized by widespread, intermittent bursts of high-amplitude activity (B). Compared with healthy controls at 6 months (C), West syndrome is associated with chaotic and disorganized high-amplitude activity with mixed-frequency components (hypsarrhythmia, D). All EEGs are shown in average referential montage.

Subject characteristics are detailed in Table 1. Two control subjects were identified from routine clinical service in a tertiary pediatric hospital providing specialist regional neurophysiology services, based on their age and an EEG within normal limits without evidence of epileptiform abnormalities. Patients with Ohtahara syndrome were selected based on (a) clinical history of seizures, (b) neonatal or early infantile onset of the epilepsy, and (c) evidence of a burst-suppression pattern on standard clinical EEG. Patients with West syndrome were selected based on (a) clinical history of infantile spasms, (b) infantile onset of the epilepsy, and (c) evidence of hypsarrhythmia on standard clinical EEG. Examples of the EEGs from patients, compared with the age-matched healthy controls, are shown in Figure 1. Where identified, underlying causes for the epilepsy ranged from genetic abnormalities to localized brain lesions and brain malformations.

**Table 1.** EEG and clinical features of participants included in the analysis.

**ID**	**EEG classification**	**Age (months)**	**EEG abnormality**
1	Normal for age	2	-
2	Normal for age	6	-
3	Ohtahara syndrome	1	Burst suppression
4	Ohtahara syndrome	2	Burst suppression
5	Ohtahara syndrome	2	Burst suppression
6	West syndrome	5	Hypsarrhythmia
7	West syndrome	6	Hypsarrhythmia
8	West syndrome	8	Hypsarrhythmia

All EEG recordings were performed with informed consent from the patients’ legal guardians, and as necessitated by the patients’ clinical course. Use of anonymized EEGs from the clinical database for quantitative analysis was reviewed and approved by the UCL Great Ormond Street Institute of Child Health Joint Research and Development Office.

### EEG Recordings and Preprocessing

Routine clinical EEG recordings were used for the analysis. Each patient had 19–21 scalp electrodes placed according to the International 10–20 system. Recordings lasted for up to 30 min during task-free resting with the subjects’ parent or guardian. Data were recorded with a sampling frequency of 256 or 512 Hz and Butterworth bandpass filtered to a 1–80 Hz frequency band for visual analysis.

For each individual subject a total of five artefact-free 10-s segments of EEG were selected for further analysis, excluding periods of visually apparent deep sleep. No distinction was made for light sleep and awake segments in the EEGs where no obvious electrographic sleep architecture was appreciated on visual analysis. Because of the severity of the EEG abnormalities, sleep stages were not apparent for some of the patients.

Further analysis was performed on these data segments, each filtered to six different standard EEG frequency bands: broadband (0.1–60 Hz); delta band (1–4 Hz), theta band (4–8 Hz), alpha band (8–13 Hz), beta band (13–30 Hz), and gamma band (30–60 Hz). Quantitative analysis was performed on an average montage with each channel referenced to the overall mean scalp activity.

Selection of the EEG segments was made pseudorandomly by visually selecting artefact-free 10-s EEG epochs. No specific focus was made to include disease-specific EEG segments; however, the abnormalities for all patients were pervasive throughout the entirety of the recordings. All data segments included in the analysis can be reviewed in full in the analysis repository at Rosch (2017).

### Quantitative Network Analysis

Analysis was performed using customized scripts written by the authors running on Matlab 2016a (MathWorks, 2016), as well as the k-Wave toolbox (Treeby, Varslot, Zhang, Laufer, & Beard, 2011) for quantification of matrix sharpness and contrast. Existing routines to generate surrogate time series written by Temu Gautama and based on Schreiber & Schmitz (1996, 1999) were used to evaluate stationarity in the features described here (https://uk.mathworks.com/matlabcentral/fileexchange/4612-surrogate-data). Mixed-effects ANOVA to model repeated measure group differences were performed with routines written by Matthew Johnson, available online (https://uk.mathworks.com/matlabcentral/fileexchange/27080-mixed–between-within-subjects–anova). All scripts are available to download and free to use at doi.org/10.5281/zenodo.887316 (Rosch, 2017). Each feature was calculated separately for each frequency band.

Spatial inference from EEG is often confounded by issues regarding volume conduction and the choice of reference (Bastos & Schoffelen, 2015). The analysis presented here is specifically designed to allow inference on network *dynamics* rather than *topology* in order to identify robust quantitative features shared between patients with very different brain abnormalities (Bialonski & Lehnertz, 2013). Classical pitfalls, such as volume conduction, are not expected to introduce an artificial group difference or bias between groups, which is essential for the future potential use of this approach as a biomarker.

#### Estimating dynamic correlation pattern changes.

**Figure 2.**
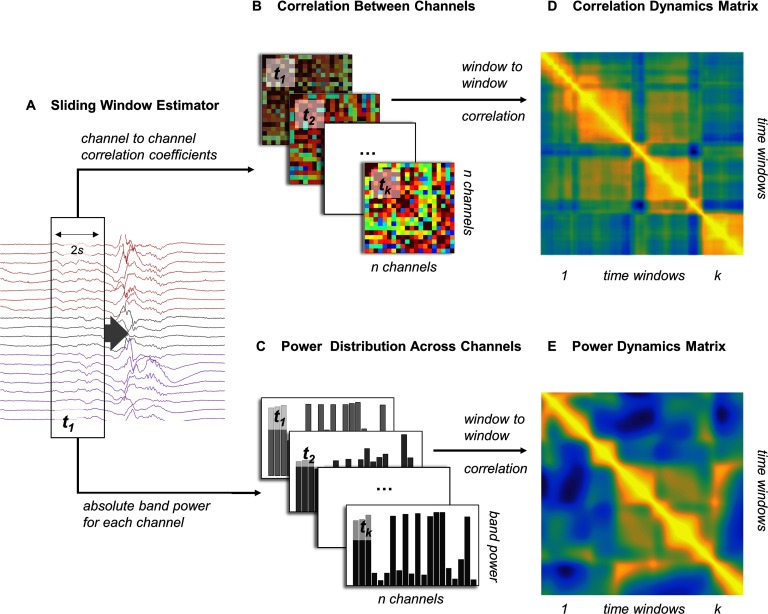
Dynamics of band power fluctuations and changing network correlations can be estimated separately. This figure summarizes the estimation of dynamic changes in the power distribution, and correlation patterns over a single 10-s window containing broadband frequencies: (A) A sliding window estimator (window length: 2*s*; step size: 1/512*s*) is used for feature extraction. (B) Channel-by-channel correlations are estimated using Pearson’s linear correlation coefficients for each time window. (C) The average power within the specified frequency band is estimated for each channel independently, resulting in a specific band power distribution for each time window. (D, E) Estimating window-to-window correlation based on these measures yields two *n* × *n* dynamics matrices describing correlation dynamics (D: CDM) and power dynamics (E: PDM), respectively.

In order to identify changes in network states, dynamic correlation patterns were estimated (summary of the analysis pipeline shown in Figure 2). For each EEG segment a sliding window approach was used to estimate dynamic changes in the patterns of correlation between channels. Starting at each sampling point between 0 and 8 s of any 10-s EEG segment, a 2-s window was extracted, yielding *k* short segments. For each short segment, pairwise Pearson’s correlation indices between individual channels were calculated, yielding a total of *k* correlation matrices that were *n* × *n* elements large each (Figure 2B, where *k* = number of steps for sliding window, *n* = number of channels).

To identify transitions between network states defined by specific scalp-electrode correlation patterns, a single *k* × *k* correlation dynamics matrix (CDM) was calculated: For this, we reshaped each of the *k* separate n × n matrices into *k* vectors of $\sum_{i=1}^{n-1}i$ unique elements from the upper triangle of the adjacency matrix (avoiding duplications as the original *n* × *n* adjacency matrix is symmetrical). The pairwise cross-correlation for all *k* vectors was then calculated to derive the full *k* × *k* CDM. This approach helps to identify temporal changes in the correlation patterns of the channel-to-channel correlation matrices (Figure 2D). Further statistical analysis was based on a single such CDM for each 10-s time window.

#### Estimating dynamic power distribution changes.

Similar to the correlation dynamics analysis, we established a related measure describing the power distribution changes over time (also shown in Figure 2). For each EEG segment, a sliding window approach was used to calculate mean band power for each channel within the respective frequency band. This yielded *k*vectors of length *n* (Figure 2C, with *k* = number of steps for sliding window, *n* = number of channels). A *k* × *k* power dynamics matrix (PDM) was calculated by estimating the pairwise correlation between each of the *k* power distribution vectors (Figure 2E). Further statistical analysis was based on a single PDM for each 10-s time window.

### Statistical Analysis

#### Quantifying matrix features.

**Figure 3.**
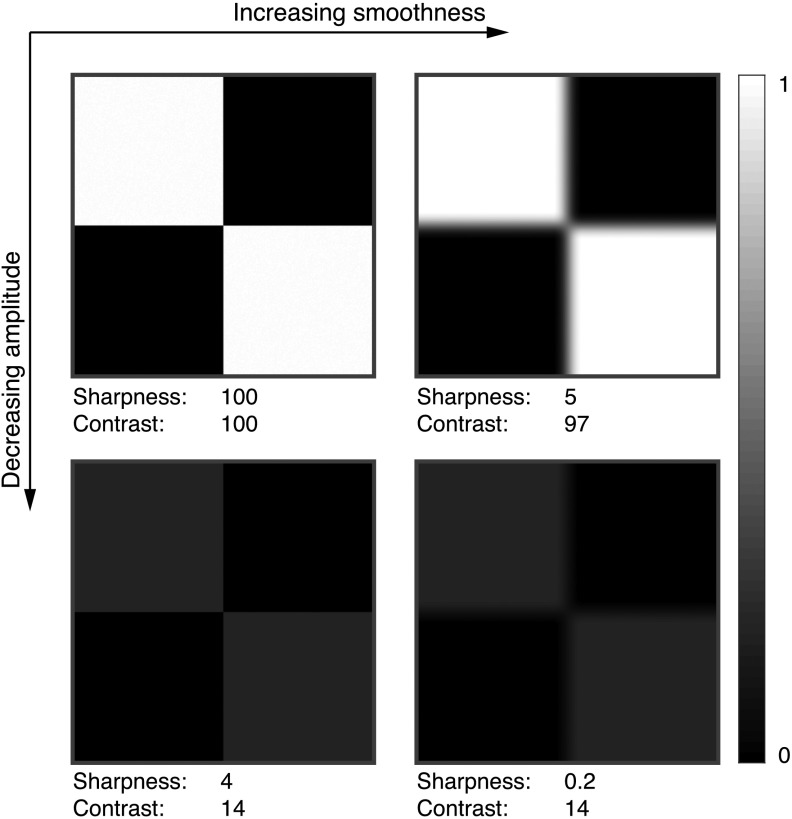
Contrast and sharpness measures encode related but different matrix features. Analysis of example matrices with different levels of Gaussian smoothing (left vs. right panels), and different signal amplitudes (top vs. bottom panels) shows differential sensitivity of the measures employed (normalized values shown): Contrast changes mainly with the amplitude of the regional signal differences, while sharpness is affected by both amplitude and smoothness modulations.

To quantify features in the dynamics matrices, a set of scalar measures was calculated for each dynamics matrix; these are summarized in Table 2. Briefly, they include the matrix mean (i.e., dynamic correlation averaged in time); matrix contrast; and matrix sharpness (defined as the Brenner Operator; Treeby et al., 2011). Contrast and sharpness measures are derived from image analyses, and in this context they represent measures describing the transition between correlated network states in time. They are differentially sensitive to regional amplitude differences (where contrast is more robustly sensitive) and smoothness of transition between states (where sharpness is more robustly sensitive), as illustrated in Figure 3.

**Table 2.** Measures used for quantification of dynamics matrix features.

Mean values	$\underset{x,y}{\sum }f_{x,y}\;/\;k^{2}$
Contrast*	$\underset{x,y}{\sum }{\left(f_{x,y}-\;f_{x+1,\;y+1}\right)}^{2}$
Sharpness (Brenner operator)**	$\underset{x,y}{\sum }{\left(f_{x+2,y}-\;f_{x,y}\right)}^{2}+{\left(f_{x,y+2}-\;f_{x,y}\right)}^{2}$

* Derived indirectly from the gray-level co-occurrence matrix.

** Modified from Treeby et al. (2011).

*f*_*x*,*y*_ = *f*(*x*, *y*), the scalar value of the matrix at column *x*, row *y*. *k* is the number of rows in the matrix.

#### Testing for stationarity and statistical differences.

To evaluate whether the dynamics matrix features reflect nonstationary processes, they were statistically evaluated against a set of stationary surrogate time series of the same overall frequency and cross-correlation composition (Schreiber & Schmitz, 1999). For each individual time window included in the analysis, the following analysis steps were performed in order to derive a stationary normal distribution of the measures illustrated above (mean values, contrast, and sharpness):

1.  A total of 100 sets of amplitude and cross-correlation-adjusted surrogate time series were generated for each 10-s window (yielding a total of 1,000–1,500 sets of EEG surrogates per group).
2.  Each set of synthetic time series were then analyzed using the same sliding window analysis applied to the original datasets, thus deriving 100 surrogate sets of the measures described above for each 10-s time window analyzed.
3.  Z-scores were calculated for each measurement derived from the empirical time series, based on the distribution of synthetically generated surrogate measurement sets.

If a dynamics matrix measure reflects only stationary processes caused by random fluctuations around steady-state frequency distributions, empirical values are expected to fall within the normal distribution of the surrogate data, that is, roughly within the − 2 to 2 z-score interval. We also used the z-score normalized data to test for differences between individual measures using two-sided *t* tests.

#### Clustering based on network dynamics.

The approach taken above yields several distinct measures for each EEG time window: three dynamics matrix measures (mean, contrast, sharpness) for two different matrix types (CDM, PDM), for six frequency bands (broadband, delta, theta, alpha, beta, gamma) resulting in a total of 36 feature measures for each EEG window. To identify dynamic network measures that capture discriminatory features between the EEG abnormalities analyzed here, we (a) measured how well they can be used to classify individual EEG segments into distinct groups, and (b) used a subset of the highest ranking measures to automatically identify clusters within the data using machine-learning approaches.

For each measure we identified thresholds that optimize the classification of EEG windows into three clusters corresponding to the three participant groups (i.e., Ohtahara syndrome, West syndrome, and healthy controls). To that effect, the purity of the classification, *P*, resulting from a set of two threshold parameters was maximized using a simulated annealing approach. *P* ranges from 0 (no element is correctly categorized) to 1 (all elements are correctly categories) and is calculated as follows:

$$P\left(\Omega ,\mathbb{C}\right)=\frac{1}{N}\underset{k}{\sum }\underset{j}{\text{max}}|\omega_{k}\cap c_{j}|\;,$$

where Ω = {*ω*_1_, *ω*_2_, …, *ω*_*k*_} is the set of estimated clusters, and ℂ = {*c*_1_, *c*_2_, …, *c*_*j*_} is the set of given classes.

The maximally achieved value for *P* was recorded for each measure and used to rank the 36 individual dynamics measures according to how well they can be used to cluster EEGs into patient groups. As a second step, we then used subsets of the high-ranking dynamics measures to automatically cluster the EEGs into different groups using *k*-means clustering. This approach partitions a dataset into a set of *k* clusters automatically, given a set of observations. We use this approach to quantify how well the measures we identified in the first steps can be used to categorize 10-s segments of EEG into the appropriate participant categories (using the purity measure *P*), and how well they distinguish between normal and abnormal (Ohtahara and West syndromes combined) categories (using sensitivity and specificity estimates). This does not aim to assess individual measures in terms of their diagnostic accuracy, but to quantify how much information about the original classification based on full EEG recordings is retained in this low-dimensional feature space.

## RESULTS

### Correlation and Band Power Dynamics

**Figure 4.**
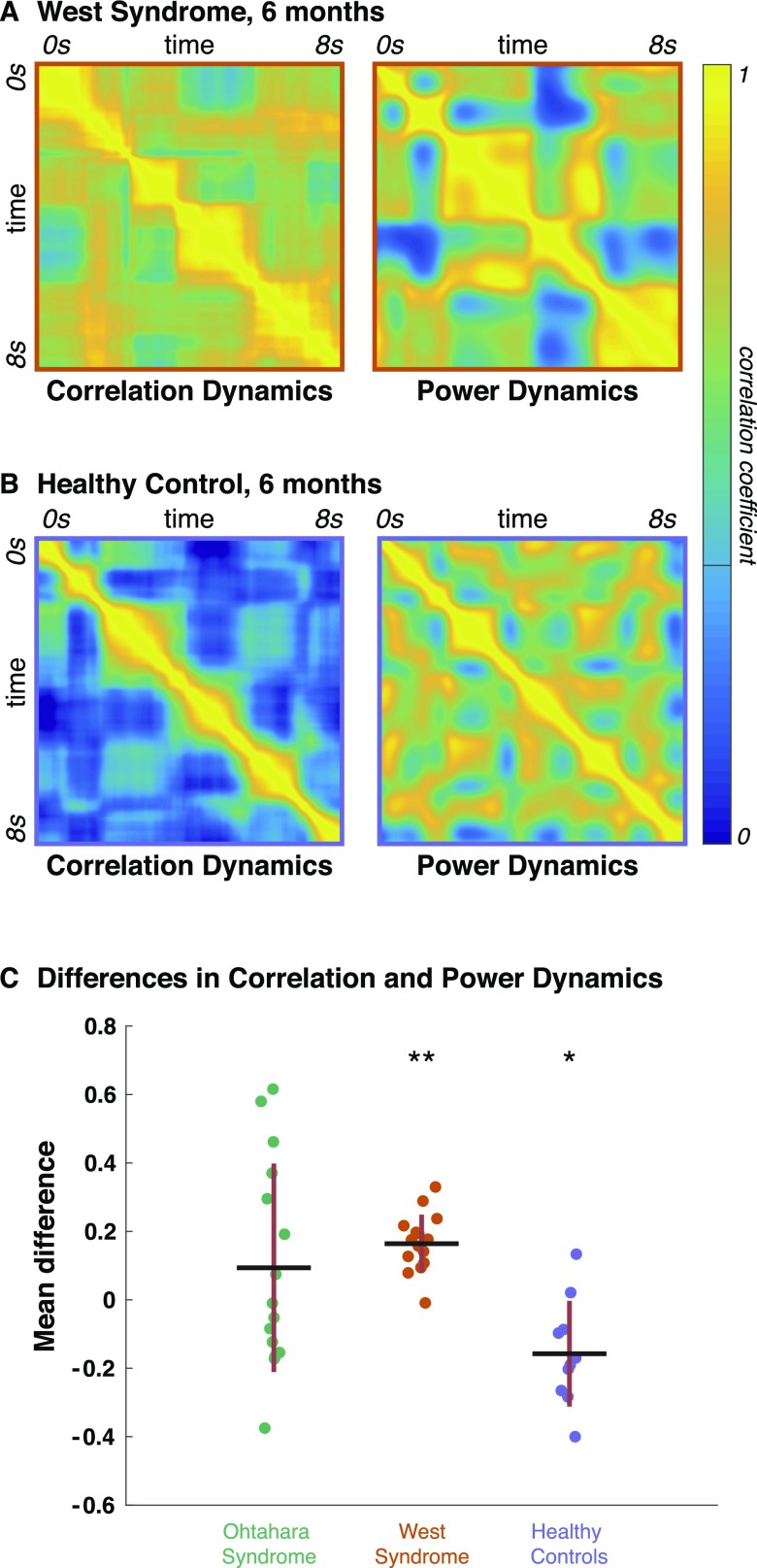
Network correlation states and band power distribution show different recurrence patterns in time. The dynamics matrices show recurrent correlation or band power distribution patterns in time. (A) Shows CDM and PDM for a single 10-s EEG segment of a patient with West syndrome containing broadband frequencies. Both show high correlation values outside of the leading diagonal (i.e., between different time segments). (B) In healthy controls, high between-time-window correlation is largely restricted to the leading diagonal in the CDM, but not the PDM, suggesting that network correlation patterns are less recurrent than band power distribution patterns for broadband frequencies. (C) Mean CDM − PDM difference values suggest that for healthy controls, but not the patient groups, recurrent band power patterns recur more across time than network correlation patterns (i.e., the CDM − PDM difference is negative).

A total of five relatively artefact-free EEG segments were selected randomly and analyzed for each participant, yielding separate CDM and PDM for each segment and each frequency band. Across all subjects and segments there are visible differences in the temporal dynamics of correlation patterns and band power distribution patterns (shown in Figure 4A–B), which can be quantified in the difference between the two matrices: if band power distribution and channel-to-channel correlation followed the same dynamics, the differences would be expected to center around 0. The mean of the CDM − PDM difference is shown for each time window in the analysis in Figure 4C. This suggests that in healthy controls, PDM values are overall higher than CDM values, which is also seen in Figure 4B. In both patient groups, there is more temporal cross-correlation in network correlation states than in the band power distribution, resulting in positive mean difference values (which only achieve significance in the West syndrome patients).

**Figure 5.**
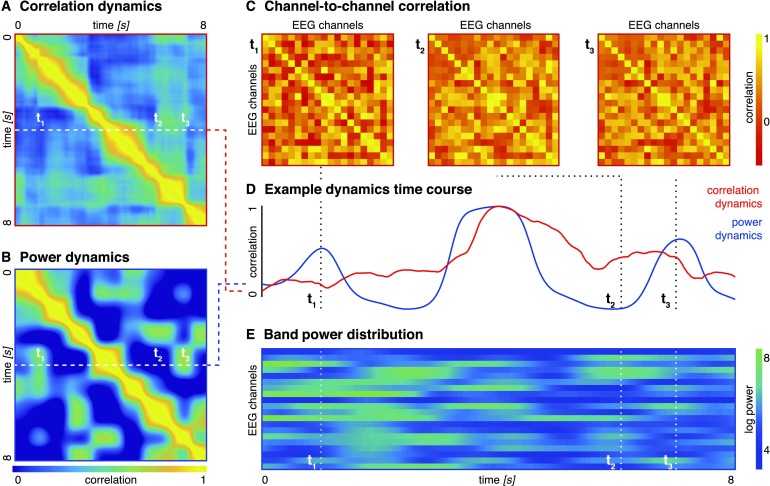
Dissociation of band power and network correlation dynamics. (A) Shows the CDM for a single 10-s EEG broadband segment in healthy control aged 2 months. (B) Shows the PDM for the same EEG segment. (C) Channel-to-channel correlation patterns are shown for three separate time windows, with big differences between time point 1 and the others, and more similarities between time points 2 and 3. (D) Correlations are shown between a single time window (indicated by the dashed lines in A, B) and all other time windows based on correlation dynamics (i.e., CDM) and power dynamics (i.e., PDM). (E) Shows the band power distribution across channels over time. While there are broad similarities in the temporal trajectories of power and correlation dynamics, there are discrete instances where similar band power distribution patterns (at *t*_1_ and *t*_3_) are associated with very different correlation patterns, and vice versa (at *t*_2_ and *t*_3_).

A closer analysis of the temporal patterns underlying these differences is shown for a single healthy control EEG segment in Figure 5. Transitions between network motifs as measured through CDM or PDM show a dissociation: Observed band power distribution across the scalp may change without closely associated corresponding changes in the network correlation patterns and vice versa.

### CDM Nonstationarity and Group Differences

**Figure 6.**
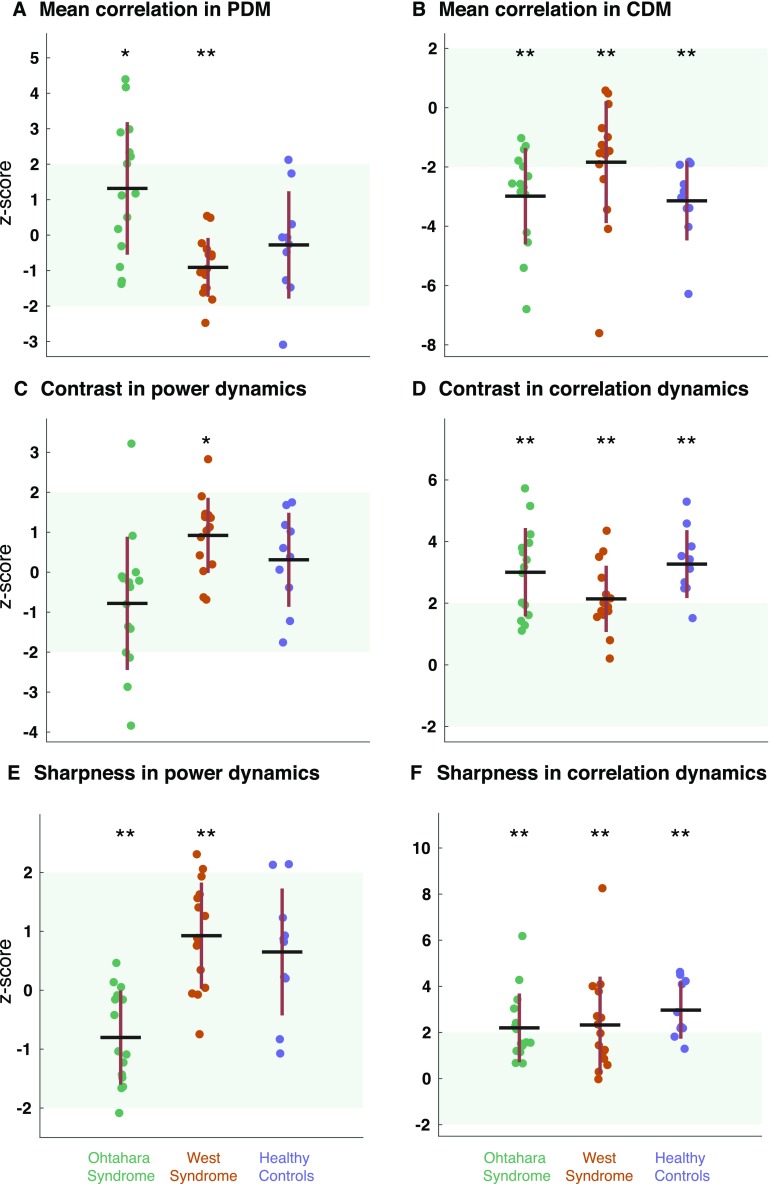
Correlation dynamics are nonstationary. Large proportions of PDM-derived measures of band power dynamics for windows containing broadband frequencies (A, C, E) fall within a 95% confidence interval derived from stationary surrogate synthetic datasets (shown as green shading), indicating that their variation at least in part may represent random fluctuations around stationary processes. Groups of measurements that differ significantly from stationary surrogate distribution are highlighted with asterisks. CDM-derived measures derived from windows containing broadband frequencies (B, D, F) are *not* fully explained by stationary correlation patterns. Red bars indicate the standard deviation around the means of the individual groups; black bars show the group mean. Asterisks illustrate results of a *t* test whether = 0: * *p* < 0.05; ** *p* < 0.01.

Randomly generated stationary time series of the same spectral composition as the empirical recordings were used to assess for nonstationarity in different measures applied to the EEG segments. For each measure, z-scores were calculated from a distribution generated based on analysis of 100 synthetic datasets for each individual EEG segment, and are shown in Figure 6. Some of the dynamics measures derived from the PDM can be explained as random fluctuations around a stationary distribution, while all CDM-derived measures differ significantly from the stationary distributions; that is, they show nonstationarity.

**Figure 7.**
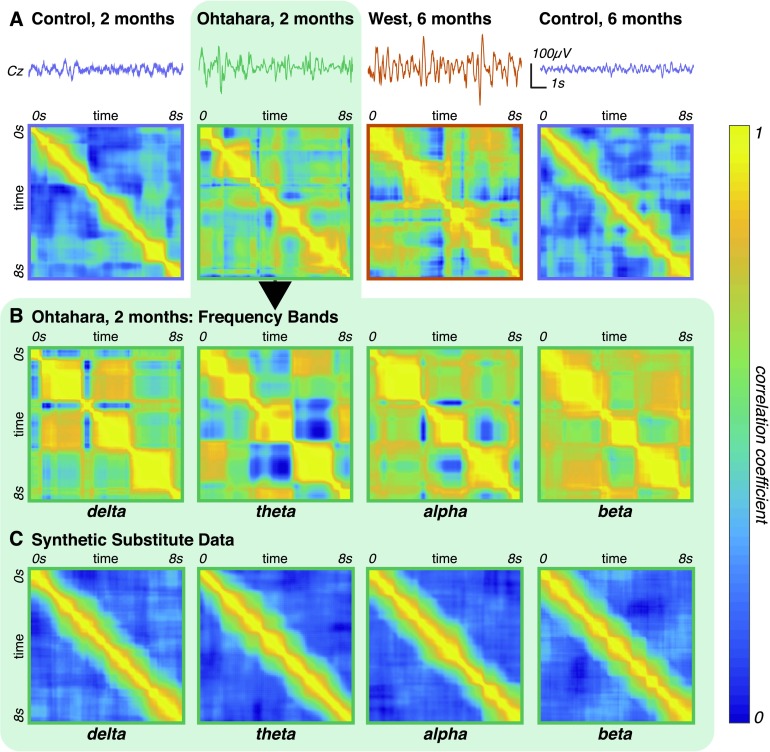
Recurrent and persistent motifs of network correlation states are more apparent in both patient groups. (A) Broadband Cz-electrode time series and CDMs are shown for single 10-s EEG segments for representatives of both Ohtahara and West syndrome cohorts and healthy age matched controls. (B) CDMs derived from bandpass filtered data for the EEG segment from the Ohtahara patient show distinct dynamics patterns for each frequency band. (C) CDMs derived from stationary surrogate data are largely restricted to the leading diagonal, thus indicating few persistent or recurrent network patterns in any of the frequency bands.

There are significant group differences in the mean correlation in the CDMs, but not the PDMs: Mixed-effects ANOVA for repeated measures − PDMs, between subjects factor F(2) = 0.41, *p* = 0.68; CDMs, between subjects factor F(2) = 6.74, *p* = 0.038. Mean correlations (+/− standard error around the mean) in CDMs of both patient groups are higher than in the healthy controls (Ohtahara syndrome: 0.59 (+/− 0.05), West syndrome: 0.75 (+/− 0.02), Healthy controls: 0.44 (+/− 0.02). The nonstationarity is expressed across frequency bands as persistent motifs, as shown for an example segment of the EEG of a patient with Ohtahara syndrome in Figure 7.

### Network Dynamics in EIEE Patients

**Figure 8.**
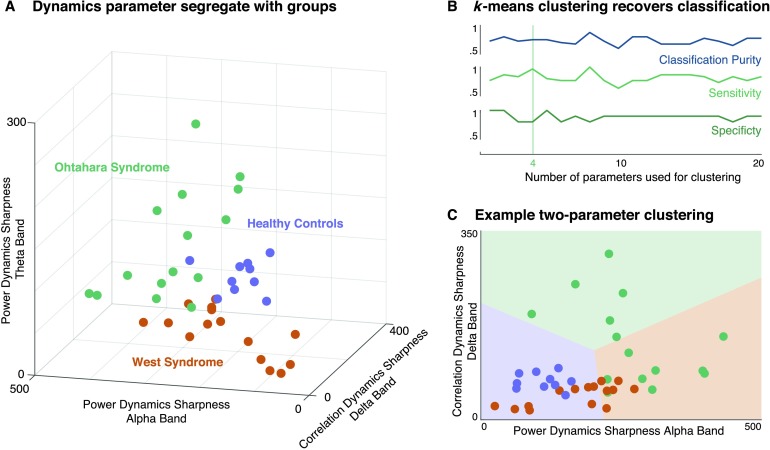
Clustering using dynamics measures separates patient groups. (A) Dynamics matrix measures can be used to visualize clustering of the EEG segments in a three-dimensional feature space. Here all EEG segments are mapped onto the three most discriminatory dynamics measures, producing distinct clusters in this three-dimensional feature space. (B) Classification purity, as well as sensitivity and specificity of classifying patients and healthy controls in separate groups, is shown for a range of parameters included in the automated clustering. (C) One example clustering solution is shown using only the two highest ranking parameters with limited separation between West syndrome and healthy control EEGs.

A complete set of dynamics measures derived from both CDM and PDM were collated for each EEG segment, resulting in 36 measures (3 measures × 2 matrix types × 6 filter bands) per EEG segment. For each individual value, thresholds were identified that could be used to separate the data into three clusters that best reproduced the original participant groups (Ohtahara syndrome, West syndrome, and healthy controls). Table 3 shows the 10 highest ranking measures based on maximum purity achieved after automatic threshold optimization. Several of these variables can be used in conjunction to map out distinct groups’ distributions in a low-dimensional feature space. As an example, Figure 8 shows each individual EEG segment mapped onto the three highest ranked dynamics measures from Table 3. In order to verify how well these measures separate distinct subgroups, we evaluated results from a *k*-means automatic clustering algorithm based on increasingly large subsets (range: 1 to 20 parameters) of the dynamics measures. Results of this unsupervised clustering approach were then compared with the known disease category, using overall classification purity, as well as sensitivity and specificity of separating healthy controls segments from patients (Figure 8B). An example of such a clustering is shown for just two parameters in Figure 8C. Using the top-ranking four parameters, this approach can reach a classification purity of 75.0%, with a disease classification sensitivity of 96.7% and a specificity of 80.0% (Figure 8B).

**Table 3.** Rank of dynamics measures based on clustering ability.

**Rank**	**Measure**	**Matrix type**	**Filter band**	**Purity**
1	sharpness	PDM	alpha	0.725
2	sharpness	CDM	delta	0.700
3	sharpness	PDM	theta	0.700
4	sharpness	CDM	all	0.675
5	sharpness	CDM	theta	0.625
6	mean	CDM	all	0.600
7	mean	CDM	all	0.600
8	mean	PDM	theta	0.600
9	contrast	CDM	theta	0.600
10	sharpness	CDM	alpha	0.600

## DISCUSSION

This report presents a quantitative analysis approach for identifying temporal patterns in network states in the developing brain. Using electrographically distinct epilepsy syndromes affecting most of the background EEG dynamics as illustrative cases, we show that (a) temporal correlation analysis can reveal distinct patterns from high-dimensional datasets such as EEG; (b) band power and channel-to-channel correlation dynamics can be dissociated, even in the healthy brain; (c) quantitative summary measures derived from this analysis can capture EEG differences between different electroclinical syndromes.

The novel measures introduced here describe quantitatively the temporal dynamics of whole-brain network states. Even structurally very different networks may show similarities in dynamics, which makes the measures introduced here particularly useful for identifying similarities in highly heterogeneous clinical populations. Early infantile EEGs are furthermore characterized by activity in a variety of spatial distributions (rather than the more typical posterior dominant rhythms seen in the mature EEG), indicating that analysis of the dynamics of different network patterns unfolding may be particularly informative in this age group.

### Dynamics Matrices Can Reveal Hidden Temporal Structure in High-Dimensional Data

Network-based analyses have been the conceptual basis for the recent success in resting-state fMRI in humans (Van Dijk et al., 2010). Furthermore, computational modeling approaches have enabled an understanding of the relationship of observable, macroscopic whole-brain network dynamics and local, mesoscale neuronal dynamics (Deco, Jirsa, & McIntosh, 2011). Many of the network features first described based on resting-state fMRI are also present in the analysis of EEG/MEG recordings, where they can be measured with very high temporal resolution, revealing fast, subsecond recurrent network switching (Baker et al., 2014). These fast network dynamics can be task related (O’Neill et al., 2017), and approaches similar to the one presented here have demonstrated that they are modulated by cognitive tasks, even in children (Dimitriadis, Laskaris, & Micheloyannis, 2015).

The analysis presented here is focused on identifying quantitative EEG features that (a) can show differences between pathological and healthy brain dynamics even at the level of individual subjects, and (b) can be applied to task-free resting-state EEG recordings routinely performed in a clinical setting. Given the heterogeneities in the clinical sample, the aim is not to identify specific neuroanatomical networks that reproduce between these patients, but to describe the visually apparent dynamic features using novel quantitative measures and to identify whether other, less directly visible, features can also be useful discriminators.

The dynamics matrices reveal structured patterns that capture the recurrence of correlated network states over different timescales. The dynamics of these transitions can be visually represented and, importantly, quantified using image metrics (such as sharpness and contrast) that intuitively capture features of the microstate transitions. This approach summarizes specific aspects of the multichannel, highly time resolved EEG recordings that are less amenable to standard visual analysis. While these quantitative measures may depend to some extent on the particular preprocessing steps performed on the EEG data, the identifiable group differences indicate that they are potentially useful as biomarkers to separate different disease categories.

### Band Power and Correlation Patterns Represent Different Aspects of Neuronal Circuitry Function

Correlations between channels and power distribution across channels are likely to represent physiologically separate processes that can follow distinct dynamic patterns. The spectral composition of the EEG signal (and thus its regionally specific band power distribution) is believed to result from synchronous firing within local neuronal populations; statistical correlation between distant channels is believed to be caused by direct or indirect, long-range synaptic connectivity (Buzsáki, Anastassiou, & Koch, 2012; Vanhatalo & Kaila, 2006). A difference between power- and correlation-derived network dynamics can therefore be understood to represent separately the dynamics of synaptic connectivity at the local and the network levels. The choice of cross-correlation means that when estimating the functional connectivity, the data are scaled by the total power of the time series; thus, this measure specifically reveals temporal structure in the correlations that is independent of the dynamics of power alone.

Across all participant groups, there are visible differences between dynamic patterns as derived from band power distributions (PDM) and network correlation patterns (CDM). These differences suggest that a particular band power distribution across the scalp does not correspond to network correlation patterns—that is, functional connectivity motifs—in the developing brain, both in patients and in normally developing controls. Using the approach shown here, changes in correlation patterns and band power patterns over time can be tracked separately and reveal divergent trajectories. Most of the dynamic variance contained within the PDM can be explained as random fluctuations of an overall stationary process, while correlation patterns over time differ significantly from those observed in a stationary signal, thus the product of a nonstationary process.

The first year of life is associated with a range of developmental changes affecting both local microcircuitry (e.g., synaptic pruning, neurotransmitter and receptor changes) as well as global network integration (e.g., myelinization of large white matter tracts) Chu, Leahy, Pathmanathan, Kramer, & Cash, 2014; Dehaene-Lambertz & Spelke, 2015). Identifying developmental changes in the dynamics at different neuronal scales may thus provide insight into the relationship between neurobiological changes and observed EEG patterns, particularly where there are visible EEG changes, as is the case in the EIEEs discussed here. Applied to a larger cohort, the approach illustrated here with just a small sample of pathological EEG patterns may in the future also reveal more subtle developmental patterns in the healthy developing brain by allowing quantification of network dynamic behaviors in simple network-based measures, such as the transition sharpness.

### Quantifying Abnormal Brain States in Clinical Populations

Our results suggest that as few as four measures may be sufficient to distinguish EEG segments from different disease categories with reasonable accuracy. Both Ohtahara and West syndrome are characterized by pervasive neuronal abnormalities that disrupt normal background EEG function. Their associated EEG phenotypes (i.e., burst suppression patterns and hypsarrhythmia) are readily apparent throughout most EEG segments. Thus, the analysis approach presented here is not designed to resolve diagnostic uncertainty; the distinct phenotypes included in the analysis were utilized to test whether our novel dynamics measures can reveal apparent differences in the EEG dynamics quantitatively.

As the measures derived from the dynamics matrices are quantifiable, they allow for statistical testing and the application of simple machine-learning tools. As illustrated in the approach taken here, individual measures can be ranked according to their discriminative power for clustering into disease categories, thereby identifying the features that most help differentiate different pathologies from the dynamics in the normal developing brain.

Of the 10 measures that are most distinct between different groups, 8 are measures of sharpness in either the CDMs or the PDMs—thus different patient groups and healthy controls show particular differences in their transition between different network states. The majority of the useful measures are derived from the CDMs (which are also most nonstationary), suggesting that it is specifically the temporal dynamics of the switch between discrete functional connectivity patterns that separates the groups. Notably, each of the top 10 ranking measures either were broadband measures or were restricted to the lower frequencies (delta, theta, alpha), which may be related to the window length of 2 s, as this will average out changes in high-frequency correlation patterns that are only a few cycles long. However, most of the physiological and abnormal activity we were aiming to capture is within the lower frequency ranges, which the window length appears to capture well. Both patients with Ohtahara syndrome and patients with West syndrome show abnormally high persistence of network states (Figure 4C). These observations suggest that the EIEE brain is susceptible to enter recurrent and abnormally stable functional connectivity states.

This approach complements existing computational modeling of epilepsy and seizure activity (Kramer & Cash, 2012; Schindler, Bialonski, Horstmann, Elger, & Lehnertz, 2008). The application of dynamical systems mathematical approaches to neuronal oscillators has led to the recognition of certain stereotypical seizure patterns as mathematically predictable oscillatory patterns (Izkhikevich 2000) that can be reproduced in computational simulations (Jirsa, Stacey, Quilichini, Ivanov, & Bernard, 2014). Using these in silico simulations means that we can explain the effects of genetic mutations (Peters, Rosch, Hughes, & Ruben, 2016), specific seizure responses to stimulation (Taylor et al., 2014), or seizure spread patterns (Baier, Goodfellow, Taylor, Wang, & Garry, 2012) using full generative models that bridge observable and nonobservable (hidden) spatial and temporal scales. Such approaches focus on reproducing specific features observed in empirical data—typically the particular, directly visible oscillatory patterns and their relationship to neuronal function (Breakspear, 2005; Jirsa et al., 2014). Here we offer quantitative descriptions of network-level dynamic features that appear to be modulated by both the epilepsy (i.e., persistent network states as seen in the examples in Figure 7A, resulting in the differences in Figure 4C) and the developmental stage (i.e., transition dynamics differences between the younger Ohtahara syndrome, and the older West syndrome cohort)—features that can be specifically included in future models of EIEE.

The EEG phenotypes analyzed here are distinctive and easily classified by visual analysis alone; they appear as generalized EEG features that may arise from focal brain abnormalities. Thus our analysis aimed to introduce and validate a set of simple measures of whole-brain dynamics that are not specifically dependent on individual patients’ topology, but capture the readily apparent EEG abnormalities. In addition to the power components, there are bivariate features exemplified by the cross-correlation in our analysis that are not readily accessible by visual analysis alone. While the approach is more complex than a simple stationary power analysis, it also allows us to extract frequency-band-specific dynamic patterns. Taken together, these features might allow the identification of disease-specific network states (Betzel et al., 2012). This work will be the foundation for future computational work aiming to reproduce some of these intrinsic dynamics to explore the relationship between putative neurobiological mechanisms of disease and the observed EEG patterns.

### Future Applications in the Clinic and in Epilepsy Research

First, the EEG phenomenology-based clustering approach may aid in resolving diagnostic uncertainties in neonatal EEG analysis, where the difference between abnormal patterns and normal developmental variants are more difficult to identify visually (Mathieson et al., 2016; Stevenson et al., 2015; Torres & Anderson, 1985). Correct diagnosis currently relies on the expertise of clinicians trained in pediatric (and specifically neonatal) clinical neurophysiology, who are not available in all clinical settings where accurate diagnosis of neonatal EEG patterns could be valuable. Yet there is a recent focus on improving neurological outcomes of neonatal care, which is likely to involve a significant increase in EEG recordings and monitoring in neonates at risk of seizures, requiring a corresponding scaling up of EEG interpretation capacities (Sands & McDonough, 2016; Vesoulis et al., 2014). Utilizing quantitative, computational approaches as presented here may be able to support correct diagnosis in those settings and play a role in improving clinical outcomes (Mathieson et al., 2016; Temko, Thomas, Marnane, Lightbody, & Boylan, 2011).

Second, the epilepsy syndromes under investigation here have a close relationship to developmental stages: Ohtahara syndrome typically is restricted to the neonatal or early infantile period, while West syndrome emerges in later infancy, typically between 3 and 10 months. Both share genetic causes (e.g., GABRA1 mutations; Kodera et al., 2016), and individual patients can evolve from Ohtahara syndrome to West syndrome during their development (Ohtahara & Yamatogi, 2006). Thus, understanding the neurobiological processes underlying the EEG phenotypes offers a window into the interactions between brain development and early onset pathological processes. By being able to quantify differences in network dynamics, we can identify features that are crucial in distinguishing patient groups. These quantitative features can be used as benchmarks for adapting existing models of neuronal dynamics (Baier et al., 2012; Papadopoulou et al., 2015; Proix, Bartolomei, Chauvel, Bernard, & Jirsa, 2014) to reproduce the empirical observations. With those models we will be able to test mechanistic hypotheses that link recent discoveries on the genetic basis of many of the EIEEs and our understanding of developmental processes in the infant brain, to the identifiable EEG syndromes seen in patients.

### Limitations

This study is not an attempt at testing the dynamics measures in terms of their clinical validity. The EIEE syndromes included here were deliberately chosen because of their wide-ranging impact on the background EEG and the disruption of normal brain dynamics; they are used to illustrate the validity of the method and the possibility to identify less visible group differences as a possible basis for a biomarker to separate different EIEE syndromes. Visually observed EEG differences are large; thus we have included only a small number of subjects, aiming to identify group differences with large effect sizes that are likely to be useful in future applications in clinical samples where predictive power at the single individual is required. We have used several EEG segments from the same patient. These are more likely to cluster than truly independent samples from a larger population of patients. The clustering approach therefore is not meant as a comprehensive evaluation of the diagnostic potential of this approach, but illustrates the use of a small set of quantitative measures of network dynamics in segregating EEG segments. Further evaluation of this approach will require formal analysis on a larger sample of patients.

## FUNDING INFORMATION

RER is funded by a Wellcome Trust Clinical Research Fellowship (106556/Z/14/Z). This study was supported by the National Institute for Health Research Biomedical Research Centre at Great Ormond Street Hospital for Children NHS Foundation Trust and University College Hospital.

## AUTHOR CONTRIBUTIONS

Richard E. Rosch: Conceptualization; Formal analysis; Methodology; Software; Visualization; Writing – original draft; Writing – review & editing. Torsten Baldeweg: Supervision; Writing – review & editing. Friederike Moeller: Conceptualization; Formal analysis; Writing – review & editing. Gerold Baier: Conceptualization; Formal analysis; Supervision; Writing – review & editing.

## TECHNICAL TERMS

Early infantile epileptic encephalopathy (EIEE): A group of severe epilepsies that start in early infancy and in which epileptic activity is so severe that it disrupts normal brain function and development.
Electroclinical syndrome: A set of recognizable clinical and EEG characteristics that consistently occur together, that define a distinctive and recognizable condition.
Functional network: A network description of the brain based on the temporal correlation between brain regions.
Microstate: A transient global pattern of activity measureable with EEG that appears quasistable at short, subsecond timescales.
Dynamics matrix: A correlation matrix of measures taken at successive time points that aims to visualize correlation between network-wide states over time.
Feature space: The number of dimensions required to capture features of a single observation. A feature is a quantifiable property of an observed phenomenon.
Stationarity: A time series is considered stationary if its statistical properties (e.g., mean, variance, autocorrelation) do not change over time.
Purity: An external validation for clustering algorithm that quantifies the proportion of observations that are classified correctly.
*k*-means clustering: An unsupervised clustering algorithm that partitions a number of observations into a predefined number (*k*) of clusters.
Dynamical systems: A set of variables whose change over time can be described by differential equations.
